# Roxarsone, Inorganic Arsenic, and Other Arsenic Species in Chicken: A U.S.-Based Market Basket Sample

**DOI:** 10.1289/ehp.1206245

**Published:** 2013-05-11

**Authors:** Keeve E. Nachman, Patrick A. Baron, Georg Raber, Kevin A. Francesconi, Ana Navas-Acien, David C. Love

**Affiliations:** 1 Johns Hopkins Center for a Livable Future,; 2 Department of Environmental Health Sciences, and; 3 Department of Health Policy and Management, Johns Hopkins Bloomberg School of Public Health, Baltimore, Maryland, USA; 4 Institute of Chemistry, Karl-Franzens University, Graz, Austria; 5 Department of Epidemiology, Johns Hopkins Bloomberg School of Public Health, Baltimore, Maryland, USA

**Keywords:** antimicrobial, arsenic, chicken, FDA, Food and Drug Administration, nitarsone, poultry, roxarsone

## Abstract

Background: Inorganic arsenic (iAs) causes cancer and possibly other adverse health outcomes. Arsenic-based drugs are permitted in poultry production; however, the contribution of chicken consumption to iAs intake is unknown.

Objectives: We sought to characterize the arsenic species profile in chicken meat and estimate bladder and lung cancer risk associated with consuming chicken produced with arsenic-based drugs.

Methods: Conventional, antibiotic-free, and organic chicken samples were collected from grocery stores in 10 U.S. metropolitan areas from December 2010 through June 2011. We tested 116 raw and 142 cooked chicken samples for total arsenic, and we determined arsenic species in 65 raw and 78 cooked samples that contained total arsenic at ≥ 10 µg/kg dry weight.

Results: The geometric mean (GM) of total arsenic in cooked chicken meat samples was 3.0 µg/kg (95% CI: 2.5, 3.6). Among the 78 cooked samples that were speciated, iAs concentrations were higher in conventional samples (GM = 1.8 µg/kg; 95% CI: 1.4, 2.3) than in antibiotic-free (GM = 0.7 µg/kg; 95% CI: 0.5, 1.0) or organic (GM = 0.6 µg/kg; 95% CI: 0.5, 0.8) samples. Roxarsone was detected in 20 of 40 conventional samples, 1 of 13 antibiotic-free samples, and none of the 25 organic samples. iAs concentrations in roxarsone-positive samples (GM = 2.3 µg/kg; 95% CI: 1.7, 3.1) were significantly higher than those in roxarsone-negative samples (GM = 0.8 µg/kg; 95% CI: 0.7, 1.0). Cooking increased iAs and decreased roxarsone concentrations. We estimated that consumers of conventional chicken would ingest an additional 0.11 µg/day iAs (in an 82-g serving) compared with consumers of organic chicken. Assuming lifetime exposure and a proposed cancer slope factor of 25.7 per milligram per kilogram of body weight per day, this increase in arsenic exposure could result in 3.7 additional lifetime bladder and lung cancer cases per 100,000 exposed persons.

Conclusions: Conventional chicken meat had higher iAs concentrations than did conventional antibiotic-free and organic chicken meat samples. Cessation of arsenical drug use could reduce exposure and the burden of arsenic-related disease in chicken consumers.

Arsenic-based drugs have been used in poultry production for decades (Silbergeld and Nachman 2008). Roxarsone (3-nitro-4-hydroxyphenylarsonic acid) was approved in 1944 by the U.S. Food and Drug Administration (FDA) to treat coccidiosis (a common parasitic disease in poultry), to improve feed conversion (which allows poultry to gain weight faster), and to improve meat pigmentation (Silbergeld and Nachman 2008). In 2010, industry representatives estimated that 88% of the roughly 9 billion chickens [U.S. Department of Agriculture (USDA) 2012b] raised for human consumption in the United States received roxarsone (Nachman et al. 2012). Because of concerns regarding human arsenic exposure, the practice of administering roxarsone to poultry is under question (Maryland General Assembly 2012). Arsenic toxicity is species dependent and is well established for inorganic arsenic (iAs; arsenite and arsenate). Chronic iAs exposure causes lung, bladder, and skin cancers (International Agency for Research on Cancer 2012) and has been associated with multiple noncancer health outcomes, including cardiovascular disease (Chen et al. 2011; Medrano et al. 2010; Sohel et al. 2009), type 2 diabetes (Navas-Acien et al. 2008), cognitive deficits (Wasserman et al. 2007), and adverse pregnancy outcomes (Ahmad et al. 2001).

Little is known about poultry metabolism of roxarsone. A few studies have examined total arsenic concentrations in the tissues of chickens that received roxarsone (Morrison 1969) or were assumed to have been administered the drug (Lasky et al. 2004; Wallinga 2006) (Table 1). In addition, the FDA (2011b) reported increased concentrations of iAs in chicken livers associated with roxarsone supplementation. To minimize arsenic accumulation in the edible tissues of the bird, the FDA requires a 5-day drug withdrawal period for roxarsone prior to slaughter (FDA 2012). To our knowledge, arsenic species have never been characterized in the muscle tissue of roxarsone-treated chickens. In July 2011, in response to an FDA safety evaluation (FDA 2011b), the leading manufacturer of roxarsone suspended sales in the United States. Marketing of roxarsone, however, has continued in other countries, and the manufacturer has noted that the decision to suspend roxarsone sales in the United States is under internal review (Harris and Grady 2011).

**Table 1:** Previous studies of arsenic in poultry.

Study	Analytical method	Tissue	*n*	Total arsenic (µg/kg)	iAs (µg/kg)
Morrison 1969^*a*^	NR	Liver	181	150–790	NA
Kidney	117	<100–240	NA
Muscle	181	<100	NA
Skin	144	<100	NA
Lasky etal. 2004	NR	Liver	20,559	330–430^*b *^	NA
Muscle (estimated)	20,559	NR	NA
Wallinga 2006	ICP-MS	Liver	151	ND–21.2	NA
Muscle (cooked)	90	ND–46.5	NA
FDA 2011b^*c *^	ICP-MS and IC-ICP-MS	Liver	21	275–2,940	0.1–9.1
Muscle (uncooked)	21	13.9–48.4	NA
Abbreviations: IC-ICP-MS,ion chromatography inductively coupled plasma mass spectrometry; ICP-MS, inductively coupled plasma mass spectrometry; NA, not applicable; ND, not detected; NR,not reported. ^***a***^All chickens in the study by Morrison (1969) were treated with roxarsone, and the FDA study (FDA 2011b) was an experi­mental study using roxarsone-treated and control chickens; the studies by Lasky etal. (2004) and Wallinga (2006) were not able to definitively determine which chicken samples had been treated with roxarsone. ^***b***^Annual mean concentrations from Food Safety and Inspection Service National Residue Program monitoring; the full range was not reported. ^***c***^Results are for roxarsone-treated chickens with a 5-day withdrawal period.					

Information on the iAs content of poultry meat is needed to quantify the public health burden associated with the use of arsenic-based drugs in poultry production. We conducted a market basket study of chicken meat products in the United States to estimate exposures to iAs and other arsenic species resulting from chicken consumption. As a secondary objective, we estimated cancer risks associated with consumption of chicken produced with arsenic-based drugs.

## Methods

*Sample collection*. From December 2010 through June 2011 (prior to the suspension of marketing of roxarsone), we purchased chicken breasts from 10 geographically diverse metropolitan areas across the United States (Table 2). Chicken was purchased from 5–10 grocery stores in each metropolitan area. No more than a single package of any brand of chicken was purchased from the same store, although multiple brands of chicken were purchased from the same store. We analyzed 142 chicken breast samples for total arsenic concentrations, representing 60 unique chicken brands acquired from 82 stores (47 supermarket chains). Of the purchased chicken samples, 69 were conventional, 34 were conventional antibiotic-free, and 37 were USDA Organic (hereafter referred to as “organic”). In each city, we collected 6–9 conventional chicken packages, 2–6 antibiotic-free packages, and 2–5 organic packages, except in Baltimore, Maryland, where no packages of conventional antibiotic-free chicken were purchased. Because of budgetary constraints, we measured arsenic species concentrations only in chicken samples with dry weight (DW) total arsenic concentrations ≥ 10 µg/kg, resulting in a total of 78 samples with arsenic speciation: 40 conventional, 13 antibiotic-free, and 25 organic samples.

**Table 2:** Geometric mean (95% CI) of arsenic concentrations (in µg/kg) in cooked chicken meat by sample characteristics.

Chicken sample classification	Total arsenic		Speciated arsenic						
	*n*	Total arsenic[GM(95%CI)]	*n*	iAs[GM(95%CI)]	DMA[GM(95%CI)]	Roxarsone+*n* (%)	Roxarsone[GM(95%CI)]	Unknown species+*n* (%)	Unknown species[GM(95%CI)]
All	140	3.0 (2.5, 3.6)	78	1.1 (0.9, 1.3)	3.5 (3.1, 4.0)	19 (24.3)	0.6 (0.5, 0.7)	13 (16.7)	0.3 (0.2, 0.3)
Package label
Conventional	69	3.4 (2.5, 4.5)	40	1.8 (1.4, 2.3)	2.6 (2.1, 3.1)	18 (45.0)	0.7 (0.6, 0.9)	13 (32.5)	0.3 (0.2, 0.3)
Antibiotic-free	34	2.0 (1.2, 3.0)	13	0.7 (0.5, 1.0)	4.2 (3.1, 5.6)	1 (7.7)	0.5 (0.4, 0.6)	0 (0)	—
Organic	37	3.4 (2.6, 4.5)	25	0.6 (0.5, 0.8)	4.9 (4.1, 5.9)	0 (0)	—^*a*^	0 (0)	—
Producer arsenical policy^*b *^
No known policy	46	5.6 (4.3, 7.4)	34	2.0 (1.6, 2.5)	3.8 (3.0, 4.9)	18 (52.9)	0.7 (0.6, 1.0)	13 (38.2)	0.4 (0.3, 0.5)
Conventional with prohibitory policy	57	1.6 (1.2, 2.3)	19	0.7 (0.5, 0.9)	2.6 (2.1, 3.2)	1 (6.3)	0.5 (0.4, 0.6)	0 (0)	—
Roxarsone detection
Negative	121	2.4 (2.0, 2.9)	59	0.8 (0.7, 1.0)	3.6 (3.1, 4.2)	0 (0)	—	0 (0)	—
Positive	19	10.2 (7.8, 13.4)	19	2.3 (1.7, 3.1)	3.2 (2.5, 4.0)	19 (100)	1.3 (1.0, 1.7)	13 (68.4)	0.7 (0.4, 1.0)
Metropolitan area
Atlanta, GA	13	2.2 (1.0, 5.0)	9	0.7 (0.4, 1.3)	3.2 (2.4, 4.3)	2 (25.0)	0.6 (0.3, 1.0)	2 (25.0)	0.3 (0.2, 0.8)
Austin, TX	17	3.3 (2.0, 5.6)	9	1.0 (0.5, 1.9)	2.9 (2.0, 4.4)	5 (55.6)	0.6 (0.4, 1.2)	4 (44.4)	0.4 (0.2, 0.7)
Baltimore, MD	13	4.1 (2.1, 7.9)	9	1.9 (1.2, 3.0)	3.0 (1.5, 6.0)	3 (33.3)	0.6 (0.4, 1.0)	2 (22.2)	0.3 (0.2, 0.5)
Denver, CO	17	3.9 (2.2, 6.8)	11	1.4 (0.8, 2.6)	3.5 (2.5, 4.8)	3 (27.3)	0.6 (0.5, 0.8)	0 (0)	—
Fayetteville, AR	14	3.1 (1.8, 5.5)	9	0.9 (0.4, 2.1)	2.3 (1.5, 3.7)	1 (12.5)	0.5 (0.4, 0.6)	0 (0)	—
Flagstaff, AZ	12	5.8 (3.6, 9.5)	9	1.4 (0.8, 1.8)	4.7 (3.2, 6.7)	3 (33.3)	0.7 (0.4, 1.3)	3 (33.3)	0.4 (0.2, 0.8)
Los Angeles, CA	12	3.9 (2.2, 7.0)	7	1.3 (0.6, 2.8)	4.4 (2.8, 6.8)	2 (28.6)	0.7 (0.3, 1.4)	2 (47.1)	0.4 (0.2, 0.9)
New York, NY	16	1.0 (0.4, 2.4)	5	0.8 (0.5, 1.4)	7.7 (4.1, 14.5)	0 (0)	—	0 (0)	—
San Francisco, CA	13	2.7 (1.9, 3.8)	6	0.8 (0.5, 1.2)	3.4 (2.7, 4.3)	0 (0)	—	0 (0)	—
Seattle, WA	13	2.6 (2.0, 3.4)	4	0.6 (0.5, 0.8)	3.2 (2.1, 6.7)	0 (0)	—	0 (0)	—
+, positive for arsenic species. The LODs were 1µg/kg DW for total arsenic, iAs, and DMA, and 2µg/kg DW for roxarsone; values <LOD were imputed as the corresponding LOD divided by the square root of two. ^***a***^The GMs for roxarsone and the unknown species were not calculated when concentrations for all samples were <LOD. ^***b***^Organic samples are not listed because arsenical drugs are not permitted for use in USDA Organic-certified chicken.									

Chicken samples were packed into coolers and shipped overnight by commercial carrier to Johns Hopkins Bloomberg School of Public Health. Upon receipt, chicken packages were stored at –20°C until sample preparation.

*Sample preparation*. For analysis of raw chicken samples, a single thawed chicken breast was removed from each package and sliced lengthwise into halves to create paired samples. One-half of each breast was processed raw. The other half of each breast was baked in a household kitchen oven (set at 177°C) to an internal temperature of 75°C (USDA 2012a), cooled, and frozen prior to processing. Individual raw and baked samples were homogenized separately in a blender with the addition of 50–100 mL MilliQ water (Millipore Corporation, Billerica, MA, USA) to aid blending. Blended samples were stored in sealable bags at –80°C. Between samples, the food processor and all laboratory equipment were cleaned with hot water, soaked for 30 min in a 10% nitric acid bath, and rinsed with MilliQ water.

Homogenized chicken samples were freeze-dried using a Freezone 2.5 freeze dryer (Labconco, Kansas City, MO, USA) and stored as a crumbled powder in 50-mL polypropylene tubes at 25°C. Sample weights were recorded before and after freeze-drying. Samples were shipped to the Institute of Chemistry-Analytical Chemistry, Karl Franzens University, for arsenic analyses. Samples were analyzed in a random order, and the laboratory was blinded to the type of sample (cooked vs. raw) and to paired samples.

*Arsenic analyses*. Detailed information on laboratory methods used to measure arsenic concentrations is provided in Supplemental Material, pp. 3–5 (http://dx.doi.org/10.1289/ehp.1206245). In brief, total arsenic concentrations in freeze-dried chicken meat samples were determined by inductively coupled plasma mass spectrometry (ICP-MS; Agilent 7500ce; Agilent Technologies, Waldbronn, Germany) following microwave-assisted acid digestion. For samples with DW total arsenic concentrations ≥ 10 µg/kg (*n* = 78), arsenic species were measured using high performance liquid chromatography (HPLC; Agilent 1100) coupled with ICP-MS, which served as the arsenic selective detector. The method allowed for the quantitative determination of iAs (arsenate and arsenite), dimethylarsinate (DMA), monomethylarsonate (MMA), and roxarsone. Another unknown arsenic species, perhaps a roxarsone metabolite, was also quantified in some samples in which roxarsone was detected (31 samples of 49 positive for roxarsone). Two samples contained significant amounts of arsenic eluting at the void volume of the HPLC column. We presumed that this was arsenobetaine, possibly a result of chickens being fed fish meal (Feedipedia 2013). Other unknown arsenic species were occasionally present in some of the samples, but usually at trace levels. The limit of detection (LOD) was 1 µg/kg DW for total arsenic, iAs, DMA, and MMA and 2 µg/kg DW for roxarsone. Total arsenic was detected in all samples. iAs, DMA, roxarsone, and the unknown arsenic species were detected in 100%, 99%, 27%, and 22% of the speciated samples, respectively. MMA was detected in 36% of samples, but the concentrations were low and above the quantitation limit for only 23 samples (data not shown). Given the low MMA concentrations, we dropped MMA from further analyses. For other species, values < LOD were imputed as the corresponding LOD divided by the square root of two.

Because arsenic measurements were performed on freeze-dried samples, we accounted for moisture lost during the drying process when estimating concentrations in edible meat. Sample-specific water loss dilution factors were calculated by dividing the DW sample mass by the wet weight (WW) mass less the added Milli-Q water. Then, we multiplied the DW arsenic concentration by its sample-specific water loss dilution factor to produce a WW arsenic concentration.

We used standard reference material (SRM) 1568a, rice flour (National Institute of Standards and Technology, Gaithersburg, MD, USA) to validate the method for total arsenic measurements. For quality controls, we used two in-house reference materials (rice and “low-arsenic chicken breast”) that were prepared and analyzed in quadruplicate with each batch of samples. For speciation analyses, stock solutions containing each of the following species (1,000 mg/L) were prepared in water or 1% aqueous ammonia solution (roxarsone only): arsenite and arsenate prepared from NaAsO_2_ (sodium arsenite) and Na_2_HAsO_4_·7H_2_O (sodium arsenate dibasic heptahydrate), respectively (Merck, Darmstadt, Germany); MMA prepared in-house from As_2_O_3_ (arsenic trioxide) and CH_3_I (methyl iodide) (Meyer reaction); DMA prepared from sodium DMA (Fluka, Buchs, Switzerland); and roxarsone (Vetranal, analytical standard; Sigma Aldrich, Vienna, Austria). Chromatograms for the standards and representative samples are provided in Supplemental Material, Figures S1 and S2 (http://dx.doi.org/10.1289/ehp.1206245).

Because there is currently no chicken meat reference material certified for total arsenic content, we validated the method using SRM 1568a, which has a certified arsenic content (mean ± SD) of 290 ± 30 µg/kg; our analysis of SRM 1568a showed arsenic content of 310 ± 10 µg/g (*n* = 16). We also analyzed an in-house rice reference material, obtaining a reference arsenic value of 257 ± 9 µg/kg (*n* = 105). This in-house rice reference material, used for day-to-day quality control, was analyzed with all batches of samples over the course of the study, giving the following results: mean ± SD, 252 ± 7 µg/kg (interassay coefficient of variation, 2.9%; *n* = 34, excluding a single outlier with 285.2 µg/kg). In addition, a sample of “low-arsenic chicken breast” was prepared and similarly analyzed over the course of the study, giving a mean ± SD arsenic concentration of 13.67 ± 0.99 µg/kg (interassay coefficient of variation 7.3%, *n* = 35, excluding a single outlier with arsenic at 19.1 µg/kg). The duplicate analysis of the chicken samples on 2 separate days (i.e., *n* = 4 in total) served as a check for outliers. Throughout the study (258 samples, 1,032 measurements), 6 outlier measurements were identified; in each case the sample was either remeasured or the outlier was excluded (*n* = 3 was used for those samples). Thus, the result for each chicken breast sample is the mean of four subsamples (or three in the case of an excluded outlier).

Ten samples were analyzed as lab-blinded duplicates to assess the quality of the testing and laboratory analysis. Relative percent differences (RPD) were determined for the concentrations of total arsenic and all analyzed species among the 10 pairs of samples and duplicates. The RPDs for total arsenic, iAs, DMA, roxarsone, and the unknown species were 1.9%, 12.6%, 1.1%, 7.3% and 15.8%, respectively.

*Analytical considerations*. Although several studies have involved the use of roxarsone, most of them have used anion-exchange HPLC-ICP-MS to investigate the fate of arsenicals in poultry waste (Garbarino et al. 2003; Jackson and Bertsch 2001), but few studies have investigated roxarsone in chicken meat. Dean et al. (1994) did not detect roxarsone (limit of quantification for arsenic, 0.25 ng/g) in chickens fed a roxarsone-supplemented diet with or without a withdrawal period, and Sánchez-Rodas et al. (2006) found nitarsone but not roxarsone in commercially available chicken breasts.

To test our starting hypothesis—that chickens fed roxarsone would have elevated levels of iAs—we developed an analytical method, comprising both an extraction step and HPLC, that could determine both iAs and roxarsone. We previously showed that acidic solutions suitable for extracting iAs from foodstuffs (Raber et al. 2012) were not suitable for roxarsone (Nachman et al. 2012). Concurrent with our attempts to find the most suitable extraction conditions, we explored HPLC conditions appropriate for iAs and roxarsone, and found that an anion-exchange column (PRP-X100; Hamilton Company, Reno, Nevada, USA) and a mobile phase of 20 mM malonate, pH 9.5, gave good retention and separation of DMA, MMA, iAs, and roxarsone. To simplify the arsenic speciation analysis, we tested the extraction of arsenic from chicken breast with extraction mixtures based on the HPLC mobile phase; we found that a solution of 20 mM malonic acid, pH 9.5, containing 1% of a 30% hydrogen peroxide (10 mL added to 500 mg freeze-dried chicken, and the mixture heated in a shaking water bath at 50°C for 1 hr) effected essentially quantitative extraction of arsenic from the chicken samples. Additional information on analytical considerations is available in Supplemental Material, pp. 3–8 (http://dx.doi.org/10.1289/ehp.1206245).

*Other variables*. Chicken samples were categorized by package label into three groups: organic, conventional, and conventional antibiotic-free. Under the USDA Organic certification program (USDA 2013), organic chickens are not permitted to be administered arsenic-based drugs. Conventional producers are not obligated to report arsenical drug use on package labels, and the “antibiotic-free” label does not preclude the use of arsenic-based drugs from a regulatory standpoint. Consequently, we used producer web sites, e-mail messages, and phone correspondence to determine whether companies have arsenical drug use policies. This information was used to categorize samples into a second scheme consisting of conventional samples from companies with (*n* = 59) and without (*n* = 46) stated policies prohibiting arsenical use. Speciated samples were also categorized *a posteriori* based on the presence or absence of roxarsone.

*Statistical analyses*. Statistical analyses were performed using Stata 11 (StataCorp, College Station, TX, USA). Geometric mean (GM) arsenic concentrations and 95% confidence intervals (CIs) were calculated to evaluate differences among categories of chicken samples, between samples with and without roxarsone detections, and among metropolitan areas where samples were collected. To analyze differences between matched cooked and raw samples, we performed paired *t*-tests on log-transformed arsenic data. Nonparametric Spearman’s correlation coefficients were used to assess relationships between concentrations of total arsenic and arsenic species. Statistical significance was two-tailed and set at α = 0.05.

*Risk analysis*. To estimate the increase in population cancer risks associated with use of arsenical drugs in poultry production, we calculated the difference in GM iAs concentrations between the categories of chicken products (by package label, by arsenical drug-use policy, and by positive roxarsone detection). These differences were then used to estimate lifetime average daily dose (LADD; milligrams per kilogram of body weight per day) of iAs using the formula

LADD = ([iAs] × IR)/BW, [1]

where [iAs] is the difference in iAs concentrations between sample categories (in milligrams per kilogram), IR is the per capita poultry intake rate (0.0824 kg/day) [U.S. Environmental Protection Agency (EPA) 2011b], and BW is body weight (80 kg BW) (U.S. EPA 2011a).

Cancer risk in chicken eaters was estimated by multiplying the LADD by the U.S. EPA Integrated Risk Information System (IRIS) cancer slope factor (q*) for iAs:

Risk = LADD × q* . [2]

The current cancer slope factor in the IRIS database for iAs (last revised in 1998) is 1.5 per milligram per kilogram BW per day, corresponding to skin cancer (U.S. EPA 2013). The cancer potency of iAs is currently being reassessed by the IRIS program; in an external review draft of the document, a cancer slope factor of 25.7 per milligram per kilogram BW per day has been proposed, corresponding to cancers of the bladder and lung (U.S. EPA 2010). We used this new cancer slope factor, derived from a 2010 analysis of the epidemiologic literature, in the risk analysis.

The 70-year lifetime population burden was calculated by multiplying the estimated risk by the size of the population at risk. Specifically, we assumed that 75% of the U.S. population consumes chicken, based on nationally representative data on the quantity and frequency of chicken consumption from the National Health and Nutrition Examination Survey (U.S. EPA 2011b), and we used 2011 Census data to determine the U.S. population size (311,591,917) (U.S. Census Bureau 2012).

## Results

*Arsenic species in chicken meat*. The GM for total arsenic was 3.0 µg/kg (95% CI: 2.5, 3.6) in cooked chicken samples (Table 2) and 2.4 µg/kg (95% CI: 2.0, 3.0) in raw samples [see Supplemental Material, Table S1 (http://dx.doi.org/10.1289/ehp.1206245)]. The increase in total arsenic concentrations observed in cooked samples is likely due to loss of moisture during the cooking process. Among samples with arsenic speciation (cooked, *n* = 78; raw, *n* = 65), the GMs for iAs, DMA, and roxarsone were 1.1 µg/kg (95% CI: 0.9, 1.3), 3.5 µg/kg (95% CI: 3.1, 4.0), and 0.6 µg/kg (95% CI: 0.5, 0.7), respectively, for cooked samples; and 0.7 µg/kg (95% CI: 0.6, 0.9), 2.7 µg/kg (95% CI: 2.4, 3.1), and 0.7 µg/kg (95% CI: 0.6, 0.9), respectively, for raw samples. The iAs concentrations in cooked conventional chicken samples were significantly higher than those in antibiotic-free samples or organic samples (Table 2). When conventional samples were classified according to arsenic drug-use policies, differences in iAs concentrations between groups were more apparent. The GM iAs concentrations for companies that had policies against the use of arsenical drugs (GM = 0.7 µg/kg; 95% CI: 0.5, 0.9) were significantly lower (*p* = 0.0004) than those for companies with no known policy (GM = 2.0 µg/kg; 95% CI: 1.6, 2.5).

Among cooked samples, roxarsone was detected in 19 of the 40 conventional samples and in 20 of 34 samples from producers without policies prohibiting use of arsenical drugs, compared with none of the 25 organic samples and only 1 of the 13 conventional antibiotic-free samples (from a producer with a stated arsenic-prohibiting policy).

Roxarsone was detected in cooked chicken samples from all cities except New York, New York; San Francisco, California; and Seattle, Washington; unknown species were detected in samples from 5 of the 10 metropolitan areas (Table 2). In raw samples, roxarsone was detected in samples from all cities except Seattle, and the unknown species was detected in samples from all cities except San Francisco and Seattle [see Supplemental Material, Table S1 (http://dx.doi.org/10.1289/ehp.1206245)]. We observed some differences in total arsenic, iAs, or DMA concentrations across metropolitan areas for cooked and raw samples. Cooked samples for total arsenic were higher in Flagstaff, Arizona, than in both New York (*p* = 0.001) and Seattle (*p* = 0.042). iAs concentrations in cooked samples from Baltimore, Maryland, were higher than those in Seattle (*p* = 0.04). Among raw samples, we observed a significant difference in total arsenic levels in the samples from Baltimore compared with both Seattle (*p* = 0.048) and New York (*p* = 0.044). DMA concentrations were significantly higher in cooked chicken from New York compared with Fayetteville, Arkansas (*p* = 0.02).

*Correlation among arsenic species*. In both raw and cooked samples, moderate to strong correlations were observed between iAs, roxarsone, and the unknown arsenic species, whereas correlations of these arsenic species with DMA were relatively weak (coefficients ranging from 0.11 to 0.36) (Table 3). The weak correlation between roxarsone and DMA (0.17 and 0.25 for raw and cooked samples, respectively) suggests that there may be little or no metabolic conversion of roxarsone to DMA within the chicken. The strong correlation of roxarsone with iAs (0.75 and 0.68 for raw and cooked samples, respectively) and the unknown arsenic species (0.63 and 0.52) in chicken meat suggests that at least some of those species could be roxarsone metabolites.

**Table 3:** Correlation matrices for arsenic species in raw and cooked chicken meat samples.

	Total arsenic	iAs	DMA	Roxarsone^*a*^	Unknown species^*a*^
Raw samples (*n*=65)
Total arsenic	1.00	—	—	—	—
iAs	0.62	1.00	—	—	—
DMA	0.71	0.36	1.00	—	—
Roxarsone	0.61	0.75	0.17	1.00	—
Unknown species	0.55	0.63	0.11	0.83	1.00
Cooked samples (*n*=78)
Total arsenic	1.00	—	—	—	—
iAs	0.75	1.00	—	—	—
DMA	0.65	0.33	1.00	—	—
Roxarsone	0.65	0.68	0.25	1.00	—
Unknown species	0.56	0.52	0.23	0.82	1.00
^***a***^For roxarsone and unknown species, correlation analyses were restricted to samples with a positive detection (*n*=30 for roxarsone; *n*=24 for unknown species).					

Yao et al. (2013) recently reported iAs contamination of commercial roxarsone formulations. An earlier study by Pizarro et al. (2004), which examined drinking water arsenate exposure and the resulting biotransformation and distribution of arsenic species in chicken cardiac muscle and meat tissues, suggested that chickens are capable of metabolizing iAs to DMA; these authors reported that DMA was the dominant arsenic species in the muscle tissue. Thus, DMA in chicken samples might result from metabolic conversion of iAs present as a contaminant in roxarsone formulations, or from metabolism of iAs from other sources, such as drinking water and feed.

**Figure 1:**
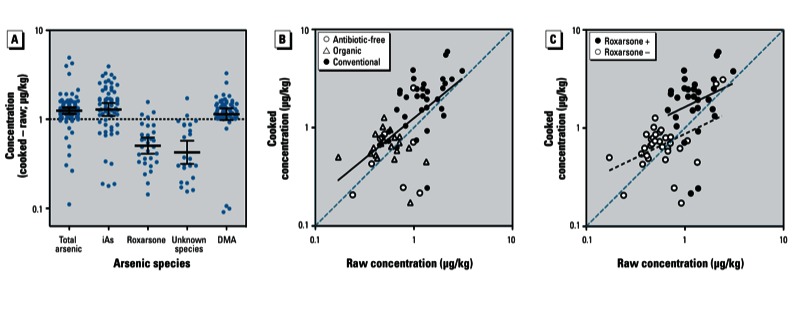
Results showing comparisons between cooked and raw sample pairs. (*A*) Means and 95% CIs for arsenic species for the difference between pairs of cooked and raw samples. (*B,C*) Scatter plots showing the effect of cooking on iAs concentration by package label (*B*) and by roxarsone presence (+) or absence (–) (*C*).

*Comparison of raw and cooked samples*. Total arsenic and iAs concentrations were significantly higher—and concentrations of roxarsone and the unknown arsenic species were significantly lower—in cooked chicken samples compared with raw samples (Table 4, Figure 1A). In Figure 1B and C, scatter plots displaying the effect of cooking on iAs concentrations are presented by package label and by roxarsone detection, respectively. [Scatter plots for total arsenic, roxarsone, DMA, and the unknown species are presented in Supplemental Material, Figure S3 (http://dx.doi.org/10.1289/ehp.1206245).] DMA concentrations were similar in raw and cooked samples. These results suggest that roxarsone and the unknown species may degrade into iAs species during cooking. We investigated this hypothesis by comparing concentrations of roxarsone, unknown arsenic species, and iAs in the same single freeze-dried raw chicken breast sample (in triplicate) before and after heating at 175°C for 30 min. In this experiment, concentrations of roxarsone and the unknown arsenic species decreased from 20 µg/kg (dry mass) and 10 µg/kg, respectively, in the raw chicken to < 2 µg/kg in the “cooked” chicken, whereas iAs increased from 11 to 42 µg/kg.

**Table 4:** Paired *t*-test comparisons of arsenic species concentrations (µg/kg) in paired raw and cooked chicken meat samples.

As species	Pairs (*n*)	Raw [GM (95%CI)]	Cooked [GM (95%CI)]	*p*-Value	Percent change
Total arsenic	102	3.0 (2.5–3.6)	3.8 (3.2–4.5)	<0.001	21.1 +
iAs	63	0.8 (0.7–1.0)	1.1 (0.9–1.3)	0.004	27.2 +
DMA	63	2.7 (2.4–3.1)	3.1 (2.5 -3.8)	0.09	12.9 +
Roxarsone^*a*^	30	1.8 (1.4–2.2)	1.0 (0.7–1.3)	<0.001	44.1 –
Unknown species^*a*^	24	1.3 (1.0–1.5)	0.8 (0.5–1.1)	<0.001	38.4 –
Abbreviations: +, increase; –, decrease. ^***a***^Analyses for roxarsone and unknown species were restricted to pairs of samples with at least 1 positive detection.					

*Risk analysis*. Compared with consumers of organic chicken, a person weighing 80 kg (U.S. EPA 2011a) who consumes an average of 0.0824 kg chicken/day (U.S. EPA 2011b) from conventional producers without policies prohibiting arsenical drug use (i.e., with a GM iAs concentration of 0.002 mg/kg) would ingest an additional 0.115 µg iAs/day, resulting in a lifetime average daily dose of 1.44 × 10^–6^ mg/kg BW/day. Based on the U.S. EPA’s proposed cancer slope factor for iAs of 25.7 per milligram per kilogram per day (U.S. EPA 2010), the average daily exposure at this level this would result in approximately 3.7 additional cases of bladder and/or lung cancer per 100,000 persons with lifetime exposure. When applied to the U.S. population in 2011 (U.S. Census Bureau 2012), 75% of whom are estimated to be chicken consumers (U.S. EPA 2011b), our estimates suggest that industry-wide use of arsenical drugs could result in 8,661 additional cases of cancer over 70 years, or an average of 124 cancers/year. This scenario represents the estimated increase in cancer cases if arsenical drugs are used in all domestically produced poultry compared with cases expected with no use of these drugs. The scenario does not account for consumers with high rates of chicken consumption, which have been estimated to be 3 and 6 times higher for those in the 95th and 99th percentiles of consumption compared with the typical consumer (Lasky et al. 2004). It is also important to note that this analysis relies on a proposed cancer slope factor derived based on women, who appear to be more sensitive than men with regard to bladder and lung cancers resulting from arsenic exposure (U.S. EPA 2010). Given potential exposure differentials and sensitivity to arsenic exposure, some uncertainty exists in the magnitude of the cancer burden.

## Discussion

This is the first study to characterize iAs concentrations in grocery store chicken meat produced with and without arsenical drugs. In our study, samples with detectable roxarsone (presumably representing chicken treated with arsenical drugs) had higher iAs concentrations than samples without detectable roxarsone. Roxarsone was detected in 20 of 40 conventional chicken samples, in 1 of 13 conventional antibiotic-free samples, and in none of the 25 organic samples tested. Conventional samples had higher iAs concentrations than antibiotic-free and organic samples. In addition, our results suggest that cooking chicken alters the arsenic species profile, increasing the inorganic fraction, potentially due to conversion of residual roxarsone and other uncharacterized arsenic species into arsenate and arsenite. Taken together, these findings suggest that the use of arsenic-based drugs in chicken production results in dietary exposures to iAs.

It is possible that nonpharmaceutical sources of arsenic, such as drinking water, may contribute to arsenic exposures during the production of chickens and thus play some role in residual arsenic found in meat; however, we believe that such exposures are unlikely to be fully responsible for the differences we observed between different types of samples because the rate of addition of roxarsone to animal feed is between 22.7 and 45.5 ppm (FDA 2012).

Dietary and environmental contributors to population iAs exposure include drinking water (Smith et al. 1992), rice (Meharg and Rahman 2003), and other foods (Xue et al. 2010). iAs in those sources tends to originate from naturally occurring geologic arsenic deposits (Welch et al. 2000) or environmental contamination from heavy industry or historic pesticide use (Meharg and Hartley-Whitaker 2002). In contrast, arsenical poultry drugs are deliberately administered to animals intended for human consumption. Consequently, exposures resulting from use of these drugs are far more controllable than are exposures from environmental sources. Few studies have looked specifically at the contribution of poultry to dietary arsenic intake, but an examination of individual food items as predictors of urinary arsenic in participants enrolled in a bladder cancer case–control study in Michigan found near-significant associations between dietary intake of chicken and urinary levels of total arsenic (*p* = 0.086) and DMA (*p* = 0.087) but not arsenobetaine (Rivera-Núñez et al. 2011).

The FDA has not established safety standards for iAs in foods, including rice, juice, chicken, or other foods potentially contaminated by arsenic. The FDA tolerances for total arsenic residues in poultry products (0.5 and 2 mg/kg for muscle and liver tissues, respectively) were established before 1963 (FDA 1963). In 2011, following a roxarsone feeding study, the FDA Center for Veterinary Medicine (CVM) indicated that the “CVM has determined that a safe level of inorganic arsenic [in chicken meat] is << 1 ppb” (FDA 2011b). The FDA later revised this statement, removing language suggesting a safe concentration and noting that “any new animal drug that contributes to the overall inorganic arsenic burden is of potential concern” (FDA 2011a). In our study, iAs concentrations in 94% of organic, 88% of antibiotic-free, and 93% of conventional samples from producers with policies against arsenical drug use were < 1 µg/kg. Conversely, 70% of samples of chicken meat from conventional producers without prohibitory arsenical drug policies exceeded this threshold.

Our study provides strong evidence that arsenic-based drugs used in poultry production result in increased iAs concentrations in chicken meat. Previous research by the FDA found increased iAs concentrations in the livers of chickens fed roxarsone (FDA 2011b). In addition, *Clostridium* species of bacteria present in the poultry cecum and in poultry waste have been shown to be capable of transforming roxarsone into iAs (Stolz et al. 2007). In July 2011, as a result of the FDA findings of iAs in chicken livers, the leading U.S. marketer of roxarsone suspended its sale from the domestic market, pending further study (Harris and Grady 2011). However, international sales of roxarsone—which is believed to be widely used in poultry production in countries around the world—have continued (Harris and Grady 2011). Moreover, nitarsone, another FDA-approved arsenic-based poultry drug that is similar to roxarsone, continues to be available for use in conventional poultry production in the United States (Zoetis 2013).

In the present study we used highly sensitive laboratory techniques to characterize iAs, roxarsone, and other arsenic species in chicken meat. This is the first study to quantify roxarsone residues in chicken meat; given that roxarsone is a non-naturally occurring compound, its detection in chicken meat is consistent with its deliberate use as a feed additive. Our analysis of cooked chicken meat also provides data on total arsenic concentrations and species that are directly relevant for human consumption. Moreover, we tested 63 paired raw and cooked samples to evaluate potential cooking-induced changes in the arsenic species profile.

Because of budgetary constraints, we speciated arsenic only in samples with total arsenic concentrations >10 µg/kg DW; thus, uncertainty remains with regard to the species profile below this threshold. Despite this limitation, we had sufficient power to detect statistically significant differences in arsenic species across sample classifications. In this study, we measured arsenic only in chicken breast meat. Additional measurements are needed to estimate arsenic concentrations in other chicken tissues such as skin, wings, thighs, and legs. There is also some uncertainty associated with the selected cancer slope factor, which has yet to be finalized. This selection, however, does reflect the U.S. EPA’s latest interpretation of the epidemiologic evidence (U.S. EPA 2010) and corresponds to internal, rather than skin, cancers.

## Conclusions

The present study provides strong evidence that the use of arsenic-based drugs contributes to dietary iAs exposure in consumers of conventionally produced chickens. Our findings suggest that eliminating the use of arsenic-based drugs in food animal production could reduce the burden of arsenic-related disease in the U.S. population.

## Correction

In the original manuscript published online, several arsenic concentrations were incorrectly given in micrograms per gram. In “Methods” (fourth paragraph under “Arsenic analysis”), “285.2 µg/g,” “13.67 ± 0.99 µg/g,” and “19.1 µg/g” should have been “285.2 µg/kg,” “13.67 ± 0.99 µg/kg,” and “19.1 µg/kg,” respectively.

In addition, the lifetime average daily arsenic dose was incorrect (“Results,” last paragraph under “Risk analysis”): “3.71 × 10^–5^ mg/kg BW/day” should have been “1.44 × 10^–6^ mg/kg BW/day.” These changes do not affect the results of the study.

The errors have been corrected here.

## Supplemental Material

(1.2 MB) PDF
