# Supplementary material for: Roxarsone, Inorganic Arsenic, and Other Arsenic Species in Chicken: A U.S.-Based Market Basket Sample
Source: Environ Health Perspect. 2013 May 11;121(7):818–24. doi: 10.1289/ehp.1206245 (PMC3701911; doi:10.1289/ehp.1206245)
Supplement: (1.2 MB) PDF [file ehp.1206245.s001.pdf]

SUPPLEMENTAL MATERIAL

Roxarsone, Inorganic Arsenic, and Other Arsenic Species in Chicken:

A U.S.-Based Market Basket Sample

Keeve E. Nachman, Patrick A. Baron, Georg Raber, Kevin A. Francesconi, Ana Navas-Acien, David C. Love

TABLE OF CONTENTS

Detailed laboratory methods section……………………………………………………..3

- **Reagents, standards and reference materials**
- **Instrumentation**
- **Determination of total arsenic**
- **Determination of inorganic arsenic and organic arsenic species**
- **Analytical considerations**
- **Figure S1. Anion-exchange HPLC-ICPMS chromatograms of four arsenic standards at concentrations from 0.2 to 2 μg L^{-1}**
- **Figure S2. Some representative HPLC chromatograms from standards and chicken samples**

Supplementary results…………………………………………………………………….9

- **Table S1. Geometric mean (95% CI) of arsenic concentrations (in μg kg^{-1}) in raw chicken meat by sample characteristics**
- **Figure S3. Scatterplots of total arsenic, roxarsone, DMA, and the unknown arsenic species for paired raw and cooked chicken meat samples by package label**

References……………………………………………………………………………..11

DETAILED LABORATORY METHODS SECTION

Reagents, standards and reference materials

Water used throughout this study was Milli-Q water (18.2 MΩ·cm; Millipore GmbH Vienna Austria). The following commercial products were used: nitric acid (> 69 %, p.a.) and methanol from Sigma Aldrich (Vienna, Austria); and aqueous ammonia 25 % (p. a.), hydrogen peroxide (30 %, p. a.) and malonic acid (>98 %) from Fluka (Buchs, Switzerland). For total arsenic measurements, the calibration standard was Single-Element Arsenic Standard P/N S4400-100031 (CPI International, Santa Rosa, CA, US), As in 2% nitric acid, 1000 ± 3 µg As mL^{-1}.

Instrumentation

ICPMS measurements were performed with an Agilent 7500ce, and HPLC was carried out with an Agilent 1100 series instrument (Agilent Technologies, Waldbronn, Germany). The ICPMS was equipped with a Burgener Ari Mist HP nebulizer (Burgener Research Inc, Mississauga, Canada) and a Scott double pass spray chamber. The HPLC was equipped with a binary pump, a vacuum degasser, column oven, and an autosampler with a variable 100 µL injection loop; it was connected to the ICPMS with 0.125 mm PEEK (polyetheretherketone) tubing (Upchurch Scientific, Oak Harbour, USA).

Microwave digestions were performed with an Ultraclave III (MLS GmbH, Leutkirch, Germany). Centrifugation was performed with a Hettich 2043 Mikroliter or a Rotina 420R centrifuge (Andreas Hettich GmbH & Co.KG, Tuttlingen, Germany).

Determination of total arsenic

Each sample was analyzed for total arsenic content in duplicate on two separate days (n=4) in the following manner. Portions (about 250 mg weighed with a precision of 0.1 mg) of the freeze-dried powdered samples were weighed directly into 12 mL quartz tubes, and nitric acid (2 mL) and H_2O (2 mL) were added. The tubes were transferred to a Teflon® rack of the Ultraclave microwave system and covered with Teflon® caps. After closing the system, an argon pressure of 4 x 10^6 Pa was applied and the mixture was heated to 250 °C for 30 minutes before being allowed to cool to room temperature. After mineralization, the samples were transferred to

15 mL polypropylene tubes (Greiner, Bio-one, Frickenhausen, Germany) and diluted with water to 9 mL (based on mass). Finally 1 mL of a solution containing 50 % methanol (to enhance the arsenic response) and 100 $\mu g \cdot L^{-1}$ each of Ge and In as internal standards were added to all digested samples giving a final concentration of 5 % methanol and 10 $\mu g \cdot L^{-1}$ of Ge and In. All standards for total arsenic determinations were prepared with 20 % nitric acid and also 5 % methanol for matrix matching with the digested samples. The arsenic concentrations in the digests were determined by ICPMS using helium as collision cell gas for removing polyatomic interferences from argon chloride ($^{40}Ar^{35}Cl$ on ^{75}As), and a ten point calibration curve in the range 0.02 – 10 µg arsenic L^{-1}.

Determination of inorganic arsenic and organic arsenic species

Extraction of arsenic species

We extracted the arsenic species with 20 mM malonic acid at pH 9.5, an extractant that essentially matched the HPLC mobile phase. Additionally, the extractant solution included a small amount of hydrogen peroxide to convert all arsenite to arsenate. The advantages of converting all arsenite to arsenate prior to HPLC have been discussed.(Raber et al. 2012) The influence of H_2O_2 on the extraction and stability of roxarsone was tested by spiking roxarsone (0.2 µg arsenic) to duplicate portions (250 mg) of "roxarsone-free" chicken breast samples, which were then extracted with 5 mL of a solution of 20 mM malonic acid pH 9.5 containing 0 %, 1 % or 2 % of a 30 % hydrogen peroxide solution for in a shaking water bath for 60 minutes at 50 °C. Roxarsone (0.2 µg arsenic) without the chicken sample was treated in an identical manner, and the products from both sets of experiments were investigated by HPLC-ICPMS. The results of this experiment (see below) confirmed that roxarsone extraction was not affected by adding H_2O_2.

Chicken samples were extracted in duplicate in the following manner. A portion (about 500 mg weighed with a precision of 0.1 mg) of the freeze-dried powdered samples was weighed into 50 mL polypropylene tubes, and a solution (10 mL) of 20 mM malonic acid adjusted to pH 9.5 with aqueous ammonia containing 1 % (v/v) hydrogen peroxide solution (30 % v/v) was added. Samples were extracted by placing the tubes in a GFL-1083 shaking water bath (Gesellschaft für Labortechnik, Burkwedel, Germany) at 50 °C for 60 minutes. After cooling to

room temperature, the extracts were centrifuged for 15 min at 4700 rcf. The supernatant was filtered through syringe filters (0.22 µm) directly into HPLC vials.

HPLC-ICPMS analyses

Arsenic speciation analyses were performed only on those samples with total arsenic content ≥ 10 µg arsenic kg^{-1} of freeze dried sample (55% of the total sample set). HPLC separations were performed under anion-exchange conditions at 40 ºC with PRP-X100 columns (Hamilton Company, Reno, Nevada, USA) and a mobile phase of malonic acid (20 mM to 100 mM at pH 9.5, adjusted with aqueous ammonia). Full details are provided in the Figure legends to the example chromatograms (Figures S1 and S2 below). For all samples, each of the duplicate extracts was analyzed under the anion-exchange conditions; differences between samples were typically < 6 % for DMA and inorganic arsenic, and <10% for roxarsone for the duplicates; the presented data represent means of the duplicates.

The signals at *m/z* 75 (^{75}As, $^{40}Ar^{35}Cl$) and *m/z* 77 ($^{40}Ar^{37}Cl$, to ascertain possible chloride interference on *m/z* 75) were monitored using a dwell time of 300 ms. An optional gas (1 % CO_2 in argon) was introduced through a T-piece connecting the spray chamber and the torch to enhance the arsenic response, as first reported for selenium (Kuehnelt et al. 2006). The data evaluation was carried out with chromatographic software G1824C Version C.01.00 (Agilent, Waldbronn, Germany). The quantification was done by external calibration against standard arsenic species based on peak areas. For the chicken meat samples, we obtained recoveries mostly between 80-120%. The mean (SD) was 102 (16) %.

Analytical considerations

There have been several studies dealing with the use of roxarsone in the poultry industry, most of which have used anion-exchange HPLC-ICPMS to investigate the fate of arsenicals in poultry waste (Garbarino et al. 2003; Jackson and Bertsch 2001). Surprisingly, there have been few studies investigating roxarsone, or other arsenic-containing growth enhancing chemicals, in chicken meat. Dean et al (1994) (Dean et al. 1994) did not detect roxarsone (limit of quantification 0.25 ng arsenic g^{-1}) in chickens fed on a roxarsone-supplemented diet with or without a withdrawal period, and Sánchez-Rodas et al. (2006) (Sánchez-Rodas et al. 2006) found nitarsone but not roxarsone in commercially available chicken breasts. The two studies used

very different extraction methods, the first employing trypsin digestion while the second used aqueous methanol.

The focus of our study was the determination of inorganic arsenic in chicken meat. Our starting hypothesis was that chickens fed roxarsone would have elevated levels of inorganic arsenic in the meat, regardless of the withdrawal period, because of metabolism of roxarsone (Stolz et al. 2007). Thus, an analytical method, comprising both an extraction step and HPLC, that could capture both inorganic arsenic and roxarsone was essential for the study. In a previous study we had shown that acidic solutions were efficient for extracting inorganic arsenic from foodstuffs (Raber et al. 2012), however, these conditions were not suitable for roxarsone (Nachman et al. 2012). Concurrent with our attempts to find the most suitable extraction conditions, we explored HPLC conditions appropriate for inorganic arsenic and roxarsone. The previously reported malonate buffer system at pH 5.6 proved excellent for the rapid separation of the three major arsenic species in (terrestrial) foods, namely inorganic arsenic, monomethylarsonate (MMA) and dimethylarsinate (DMA)(Raber et al. 2012). At this pH, however, roxarsone is too strongly retained leading to unacceptably long retention times. By increasing the pH of the mobile phase to 9.5, good retention and separation of DMA, MMA, inorganic arsenic, and roxarsone was achieved.

To simplify the arsenic speciation analysis, we tested the extraction of arsenic from chicken breast with the HPLC starting mobile phase, namely 20 mM malonic acid buffer at pH 9.5, but obtained very low recoveries (< 20%). Addition of hydrogen peroxide (1 % of a 30 % solution) to the malonate buffer, so that the extractant contained 0.3 % hydrogen peroxide, increased the recovery to about 80 %, while doubling the hydrogen peroxide content to 0.6 % did not lead to further significant improvement. The effect of the malonate/hydrogen peroxide extractant on the stability of roxarsone was then tested. When roxarsone (0.2 µg arsenic) alone was subjected to these extraction conditions, partial decomposition (10-20 %) of roxarsone to inorganic arsenic was observed. In the chicken matrix, however, roxarsone was essentially stable resulting in < 2 % conversion to inorganic arsenic at the end of the extraction procedure. These tests were performed with “low arsenic chicken” which had low inorganic arsenic content of about 6 ng arsenic g^{-1} and no detectable roxarsone (< 2 ng arsenic g^{-1}). Spiking these chicken samples with roxarsone equivalent to 200 ng arsenic g^{-1} resulted in an increased inorganic arsenic value of just 3 ng arsenic g^{-1}.

Based on these experiments, the extraction solution adopted for the chicken samples was 20 mM malonic acid at pH 9.5 containing 1 % of a 30 % hydrogen peroxide. Extraction was effected with 10 mL of this solution added to 500 mg of freeze-dried chicken sample and the mixture heated in a shaking water bath at 50 ºC for 1 hour. This achieved essentially quantitative recovery of arsenic for the chicken breast samples.

Supplemental Material, Figure S1. Anion-exchange HPLC-ICPMS chromatograms of four arsenic standards at concentrations from 0.2 to 2 µg L^{-1}.

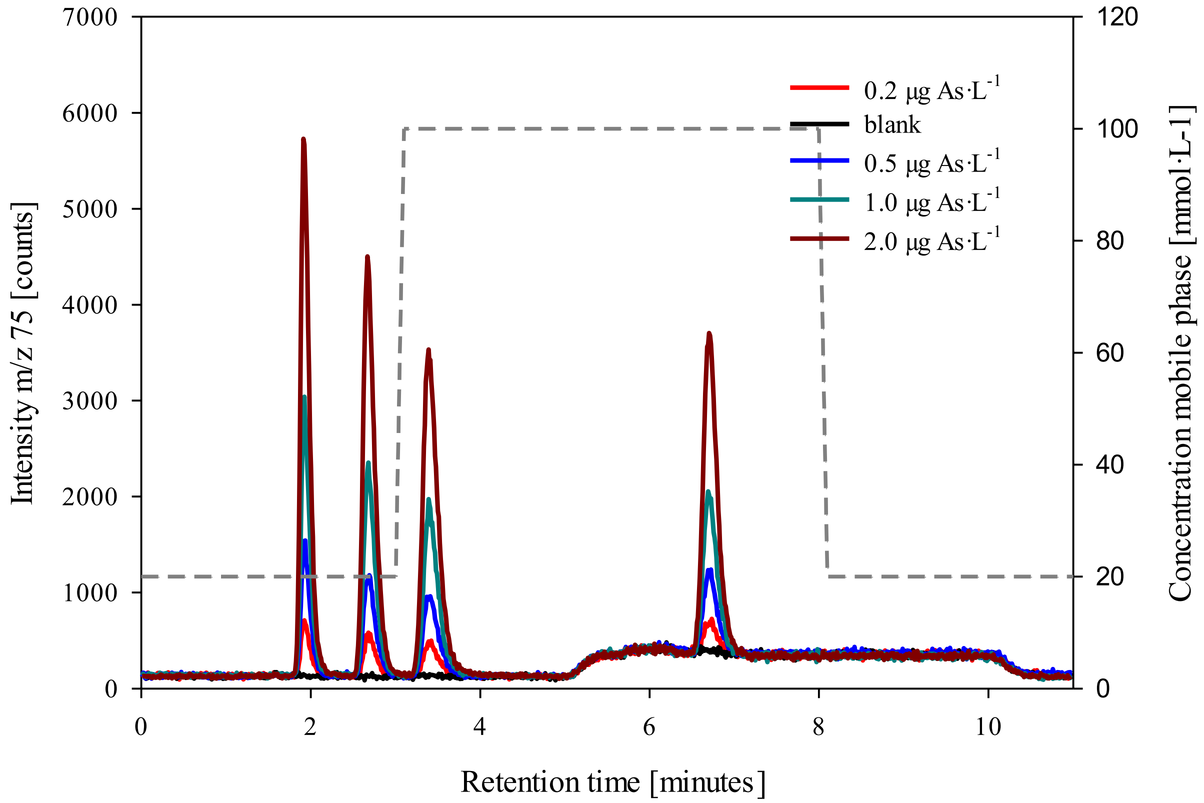

Conditions: Hamilton PRP-X100 250 mm x 4.1 mm; 10 µm particle size; mobile phase: malonic acid (20 – 100 mM) adjusted to pH 9.5 with aqueous ammonia; gradient: 0-3 min: 20 mM; 3-3.1 min: 20 mM - 100 mM; 3.1 -8 min: 100 mM; 8-8.1 min: 100 mM – 20 mM; 8.1-11 min: 20 mM; Flow rate 1.5 mL·min^{-1}; 40°C; injection volume 50 µL.

Supplemental Material, Figure S2. Some representative HPLC chromatograms from standards and chicken samples

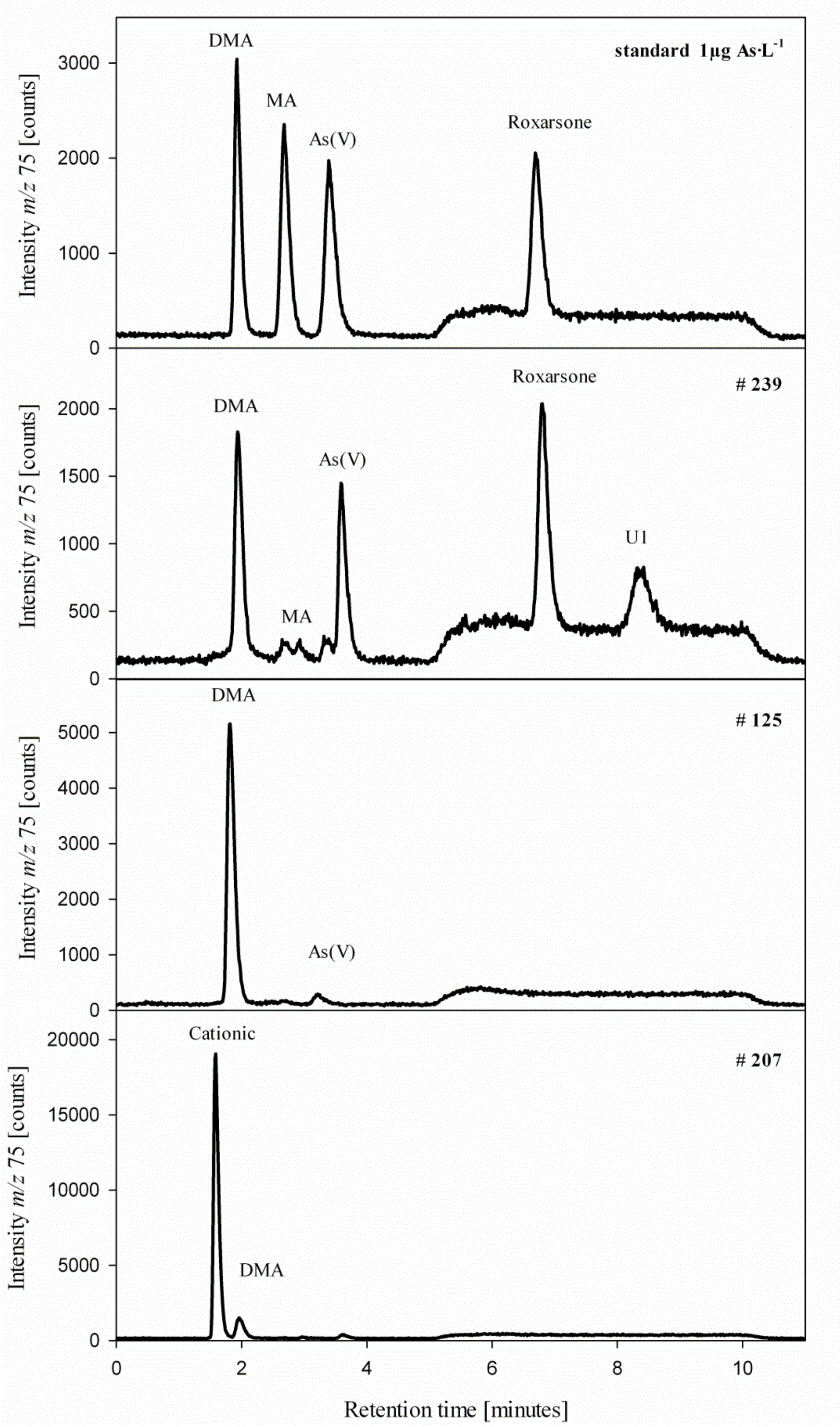

Conditions are as described in the legend to Figure S1. The cationic peak is possibly arsenobetaine.

SUPPLEMENTARY RESULTS

Supplemental Material, Table S1. Geometric mean (95% CI) of arsenic concentrations (in µg kg^{-1}) in raw chicken meat by sample characteristics[a]

	Total arsenic		Speciated arsenic							
	N	Total As	N	iAs	DMA	N (%) Roxarsone (+)	Roxarsone	N (%) Unknown (+)	Unknown Species
**All**	114	2.4 (2.0 - 3.0)	65	0.7 (0.6 - 0.9)	2.7 (2.4 -3.1)	30 (46.1)	0.7 (0.6 - 0.9)	18 (27.8)	0.2 (0.2 - 0.3)
**Package label**									
Conventional	63	2.6 (2.0 - 3.6)	37	1.1 (1.0 - 1.4)	2.2 (2.1 - 3.0)	28 (75.6)	1.2 (0.9 - 1.5)	18 (48.6)	0.2 (0.2 - 0.3)
Conventional antibiotic-free	21	1.7 (1.0 - 3.1)	9	0.5 (0.2 - 0.9)	3.2 (2.4 - 4.1)	2 (22.2)	0.4 (0.3 - 0.5)	0 (0.0)	--
Organic	30	2.6 (1.9 - 3.6)	19	0.4 (0.3 - 0.6)	3.7 (2.9 - 4.5)	0 (0.0)	--[b]	0 (0.0)	--
**Producer arsenical policy[c]**									
No known policy	41	4.1 (2.9 - 5.8)	34	1.2 (1.0 - 1.4)	2.2 (1.8 - 2.7)	27 (79.4)	1.2 (0.9 - 1.6)	17 (50.0)	0.3 (0.2 - 0.5)
Conventional with prohibiting policy	43	1.4 (0.9 - 2.1)	12	0.5 (0.3 - 0.8)	3.0 (2.4 - 3.7)	3 (25.0)	0.4 (0.3 - 0.6)	1 (8.3)	0.2 (0.2 - 0.2)
**Roxarsone detection**									
Negative	84	1.7 (1.3 - 2.1)	35	0.5 (0.4 - 0.6)	2.9 (2.3 - 3.6)	0 (0.0)	--	0 (0.0)	--
Positive	30	6.3 (5.1 - 7.8)	30	1.3 (1.1 - 1.4)	2.5 (2.1 - 3.0)	30 (100.0)	1.5 (1.2 - 1.9)	18 (60.0)	0.7 (0.6 - 1.0)
**Metropolitan area**									
Atlanta, GA	11	2.2 (1.0 - 4.0)	7	0.5 (0.2 - 1.4)	2.4 (1.8 - 3.3)	2 (28.6)	0.6 (0.3 - 1.2)	2 (28.6)	0.4 (0.1 - 0.9)
Austin, TX	14	2.3 (1.0 - 4.9)	8	0.9 (0.4 - 1.7)	2.4 (1.9 - 3.0)	6 (75.0)	1.1 (0.5 - 2.3)	4 (50.0)	0.4 (0.2 - 0.9)
Baltimore, MD	11	4.4 (2.6 - 7.4)	9	1.1 (0.7 - 1.7)	2.2 (1.1 - 4.1)	6 (66.6)	1.1 (0.5 - 2.3)	4 (44.4)	0.2 (0.1 - 0.4)
Denver, CO	11	2.9 (1.8 - 4.7)	6	1.1 (1.0 - 1.3)	2.8 (1.6 - 4.8)	5 (83.3)	0.9 (0.4 - 2.0)	2 (33.3)	0.2 (0.1 - 0.5)
Fayetteville, AK	12	2.8 (1.3 - 5.9)	8	0.8 (0.4 - 1.6)	2.1 (1.2 - 3.6)	2 (25.0)	0.5 (0.3 - 0.8)	2 (25.0)	0.3 (0.2 - 0.7)
Flagstaff, AZ	9	4.5 (2.2 - 9.3)	6	1.0 (0.6 - 1.6)	3.0 (2.0 - 4.5)	3 (50.0)	1.2 (0.4 - 3.1)	2 (33.3)	0.5 (0.3 - 1.1)
Los Angeles, CA	11	3.4 (2.0 - 5.7)	7	0.6 (0.2 - 1.4)	3.3 (2.1 - 5.2)	3 (42.8)	0.7 (0.3 -1.7)	1 (9.1)	0.2 (0.1 - 0.4)
New York, NY	15	0.9 (0.3 - 2.3)	6	0.7 (0.3 -1.7)	4.4 (2.3 - 8.6)	2 (33.3)	0.5 (0.3 - 1.0)	1 (16.7)	0.1 (0.0 - 0.7)
San Francisco, CA	10	2.5 (1.6 - 3.8)	6	0.5 (0.2 - 1.3)	3.1 (2.5 - 3.8)	1 (16.7)	0.4 (0.3 - 0.5)	0 (0.0)	--
Seattle, WA	10	1.9 (1.4 -2.5)	2	0.2 (0.1 - 0.4)	2.7 (0.1 - 10.4)	0 (0.0)	--	0 (0.0)	--

[a] Limits of detection (LOD) were 1 µg/kg DW for total arsenic, inorganic arsenic, and DMA and 2 µg/kg DW for roxarsone. Samples below the LOD were imputed as the corresponding detection limit divided by the square root of two.
[b] The geometric means for roxarsone and the unknown species were not calculated when all samples were below the limit of detection.
[c] Organic samples are not re-listed here, as arsenical drugs are not permitted for use in USDA Organic-certified chicken.

Supplemental Material, Figure S3. Scatterplots of total arsenic, roxarsone, DMA, and the unknown arsenic species for paired raw and cooked chicken meat samples by package label

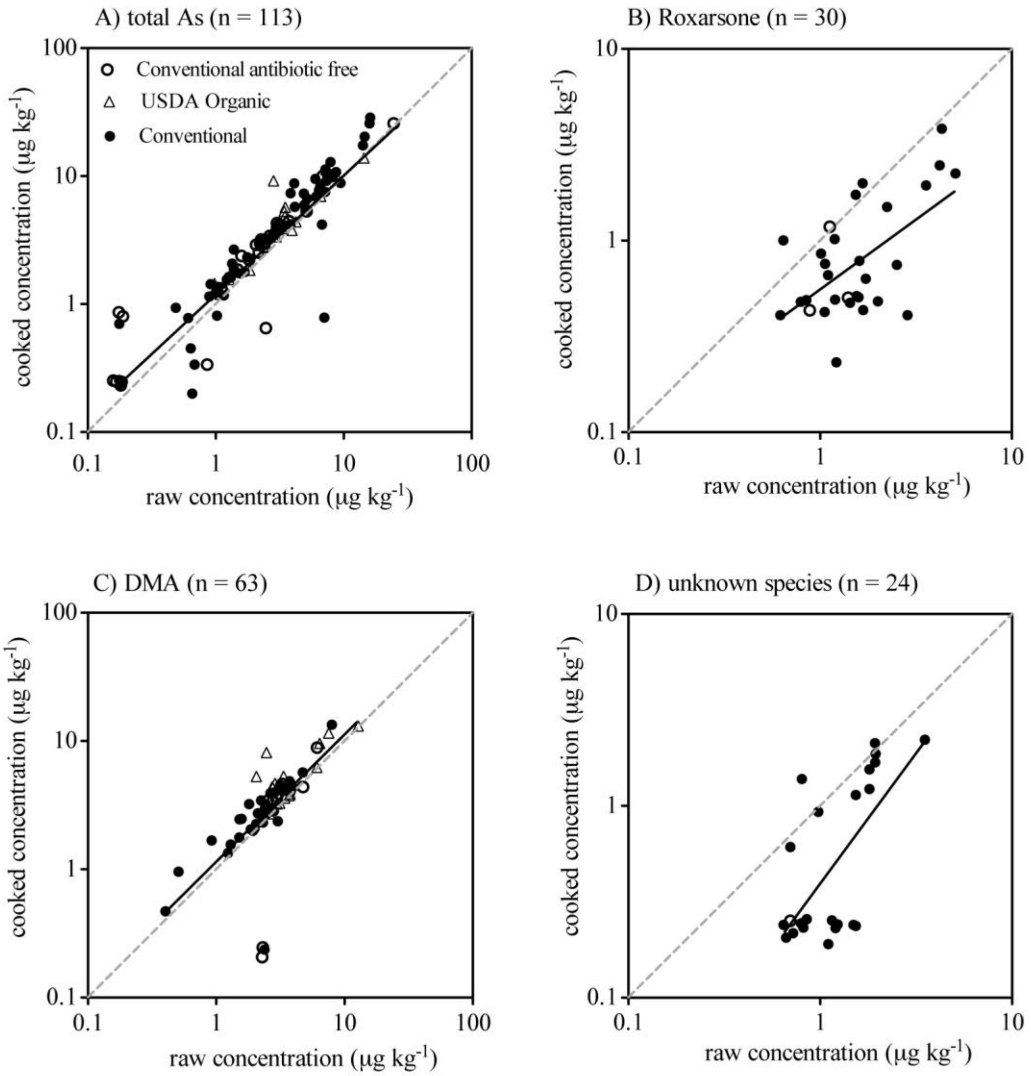

Scatterplots of concentrations of total arsenic (A), Roxarsone (B), DMA (C), and unknown species (D) in raw and cooked chicken samples. Closed circles represent Conventional chicken, open circles for conventional antibiotic free chicken, and open triangles for Organic chicken.

REFERENCES

Dean JR, Ebdon L, Foulkes ME, Crews HM, Massey RC. 1994. Determination of the growth promoter, 4-hydroxy-3-nitrophenyl-arsonic acid in chicken tissue by coupled high-performance liquid chromatography-inductively coupled plasma mass spectrometry. Journal of Analytical Atomic Spectrometry 9(5):615-618.

Garbarino JR, Bednar AJ, Rutherford DW, Beyer RS, Wershaw RL. 2003. Environmental Fate of Roxarsone in Poultry Litter. I. Degradation of Roxarsone during Composting. Environ Sci Technol 37(8):1509-1514.

Jackson BP, Bertsch PM. 2001. Determination of Arsenic Speciation in Poultry Wastes by IC-ICP-MS. Environ Sci Technol 35(24):4868-4873.

Kuehnelt D, Juresa D, Kienzl N, Francesconi K. 2006. Marked individual variability in the levels of trimethylselenonium ion in human urine determined by HPLC/ICPMS and HPLC/vapor generation/ICPMS. Analytical and Bioanalytical Chemistry 386(7):2207-2212.

Nachman KE, Raber G, Francesconi KA, Navas-Acien A, Love DC. 2012. Arsenic species in poultry feather meal. Sci Total Environ 417-418:183-188.

Raber G, Stock N, Hanel P, Murko M, Navratilova J, Francesconi KA. 2012. An improved HPLC–ICPMS method for determining inorganic arsenic in food: Application to rice, wheat and tuna fish. Food Chemistry 134(1):524-532.

Sánchez-Rodas D, Luis Gómez-Ariza J, Oliveira V. 2006. Development of a rapid extraction procedure for speciation of arsenic in chicken meat. Analytical and Bioanalytical Chemistry 385(7):1172-1177.

Stolz JF, Perera E, Kilonzo B, Kail B, Crable B, Fisher E, et al. 2007. Biotransformation of 3-nitro-4-hydroxybenzene arsonic acid (roxarsone) and release of inorganic arsenic by Clostridium species. Environ Sci Technol 41(3):818-823.
